# A review of the geometrical basis and the principles underlying the use and interpretation of the video head impulse test (vHIT) in clinical vestibular testing

**DOI:** 10.3389/fneur.2023.1147253

**Published:** 2023-04-11

**Authors:** Ian S. Curthoys, Leigh A. McGarvie, Hamish G. MacDougall, Ann M. Burgess, Gabor M. Halmagyi, Jorge Rey-Martinez, Julia Dlugaiczyk

**Affiliations:** ^1^Vestibular Research Laboratory, School of Psychology, Faculty of Science, University of Sydney, Sydney, NSW, Australia; ^2^Neurology Department, Institute of Clinical Neurosciences, Royal Prince Alfred Hospital, Camperdown, NSW, Australia; ^3^Institute of Academic Surgery, Royal Prince Alfred Hospital, Camperdown, NSW, Australia; ^4^Neurotology Unit, Department of Otorhinolaryngology Head and Neck Surgery, Donostia University Hospital, Donostia-San Sebastian, Spain; ^5^Biodonostia Health Research Institute, Otorhinolaryngology Area, Osakidetza Basque Health Service, Donostia-San Sebastian, Spain; ^6^Department of Otorhinolaryngology, Head and Neck Surgery and Interdisciplinary Center of Vertigo, Balance and Ocular Motor Disorders, University Hospital Zurich (USZ), University of Zurich (UZH), Zurich, Switzerland

**Keywords:** vHIT, vestibular, head impulse testing, SHIMPs, HIMPs, HIT, VOR

## Abstract

This paper is concerned mainly with the assumptions underpinning the actual testing procedure, measurement, and interpretation of the video head impulse test—vHIT. Other papers have reported in detail the artifacts which can interfere with obtaining accurate eye movement results, but here we focus not on artifacts, but on the basic questions about the assumptions and geometrical considerations by which vHIT works. These matters are crucial in understanding and appropriately interpreting the results obtained, especially as vHIT is now being applied to central disorders. The interpretation of the eye velocity responses relies on thorough knowledge of the factors which can affect the response—for example the orientation of the goggles on the head, the head pitch, and the contribution of vertical canals to the horizontal canal response. We highlight some of these issues and point to future developments and improvements. The paper assumes knowledge of how vHIT testing is conducted.

## Introduction

### Historical overview

**Figure 1:**
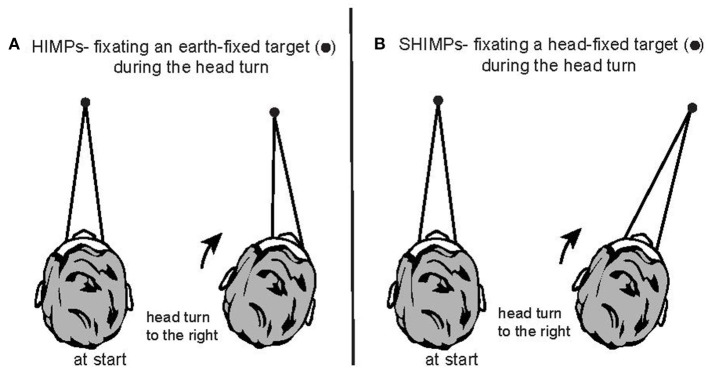
Two complementary test paradigms for using vHIT to test semicircular canal function. **(A)** In the HIMPs paradigm the person is instructed to maintain fixation on an earth-fixed target during small, abrupt, unpredictable, passive head turns delivered by the clinician. **(B)** In the SHIMPs paradigm, the head movements are identical, but the person is instructed to maintain fixation on a spot on the wall projected from a head mounted laser and which therefore moves with the head. Fixation distance is around 1 m in both cases.

In 1988 Halmagyi and Curthoys introduced a subjective test of semicircular canal function—the head impulse test (HIT)—in which the clinician observed whether there was a corrective saccade after giving the patient a brief, abrupt, passive, horizontal head turn (a head impulse) as the patient was instructed to fixate an earth fixed target (the clinician's nose) (see Figure 1A) (1, 2). Vestibular loss results in an inadequate eye movement response during the head turn so that eye velocity does not match and correct for head velocity. The consequence is that the eye is dragged, with the head, off the target so at the end of the head impulse there is an eye position error: so the patient's gaze is not directed at the target.

Consequently the patient must make a corrective saccade (a so-called “overt saccade”) in order to regain the fixation target as instructed.

HIT was validated by scleral search coil recordings of the eye movement during head impulse testing of healthy people and patients (2, 3). The key aspect of the stimulus is not the angular extent of the head impulse but the abruptness of the onset of the impulse, because the stimulus initiating the compensatory eye movement response is angular acceleration. The records of healthy subjects showed that during the head impulse the eyes execute a slow eye movement [called slow phase eye velocity (SPV)] to compensate for head turn, with the result that gaze direction is maintained on the earth-fixed target. The stimulus was a very simple head movement, but this stimulus was totally different from the usual test of semicircular canal function at that time which was to use powerful devices to deliver precisely controlled whole-body rotations to patients. So rotational testing of semicircular canals used carefully controlled low acceleration rotations (rarely above 100 deg/s/s) of a subject seated on a motorized rotating chair. In stark contrast the head impulse had acceleration values around 100 times greater than could be delivered by a motorized chair and the accelerations were comparable to those experienced in ordinary life (2,000–4,000 deg/s/s).

The apparent drawback of HIT was that the stimulus was delivered by the clinician and so varied greatly from trial to trial, in contrast to the careful control of the magnitude of accelerations delivered by motorized chairs. The very high acceleration however had the advantage of quickly silencing any contribution from the contralateral semicircular canal of the canal pair. So HIT and its later video version, the video head impulse test (vHIT), effectively tested “predominantly” the target canal (e.g., the left horizontal canal by a leftwards head impulse) and the eye movement response provided a measure of the level of function of that canal. In vHIT the emphasis was on simultaneously recording the head movement stimulus and the eye movement response and comparing the two, in full knowledge of the fact that by using human operators to deliver the stimuli there would be considerable variability in the stimulus between successive trials. This approach required analyzing the geometry of the situation and developing algorithms and programs based on a thorough geometrical understanding of the eye movement measurement procedure and a very strong emphasis on validation. We conducted many calibration trials to verify the validity of our assumptions.

With motorized chairs many clinicians carefully positioned the head so that the horizontal canal was in the plane of rotation since they presumed the rotation test was a specific test of horizontal canal function. The anatomical evidence shows that it is not possible to stimulate a single canal in isolation simply because of the anatomical fact the canals are not planar and are not mutually orthogonal (4–7). Most importantly the orientation of the labyrinth within the head varies considerably from person to person, as we discuss below [for example see Figure 4 of (7)] so the same head turn will activate different canal systems in different people. As we will show below in some individuals there is a substantial input from the posterior canals during a horizontal head turn as well as the input from horizontal canals, simply by virtue of the projection of the posterior canal into the plane of rotation. The very high acceleration of the head impulse will activate the posterior canal afferents as well as the horizontal canal afferents. This lack of specificity is important because measurement of a canal response to angular acceleration stimulation is never purely dependent on a single canal—it is mainly from the stimulated canal, but it involves all the canals (and the otoliths).

Originally the “clinical sign” of the vestibular loss was the overt saccade as detected by the clinician, but more recently the direct measure of semicircular canal function—the compensatory slow phase eye velocity (SPV)—during the head impulse, has become the standard measure of the vestibulo-ocular reflex (VOR) since the semicircular canals generate slow phase eye velocity, not saccades. Also it was difficult for many clinicians to detect the overt saccade, and as we later discovered many patients quite quickly learn to generate the corrective saccades during the head turn rather than at the end, and such “covert saccades” are not detectable by the naked eye (8). That necessitated the need for simple ways of measuring the eye movement and head movement during the head impulse, and the head-mounted, tightly fitting goggles of the vHIT recording system are such a solution.

The video head impulse test (vHIT) measures the eye movement response to a brief passive unpredictable head turn in the plane of the semicircular canals being tested—usually the horizontal canals but also more recently vertical canals. vHIT was validated by “simultaneous measures of the same eye” during testing by the “gold standard” scleral search coil procedure and video (9, 10) which showed that the vHIT video recordings matched the search coil recordings very closely. The whole test as described here is now called the HIMPs test (an acronym for head impulse test).

**Figure 2:**
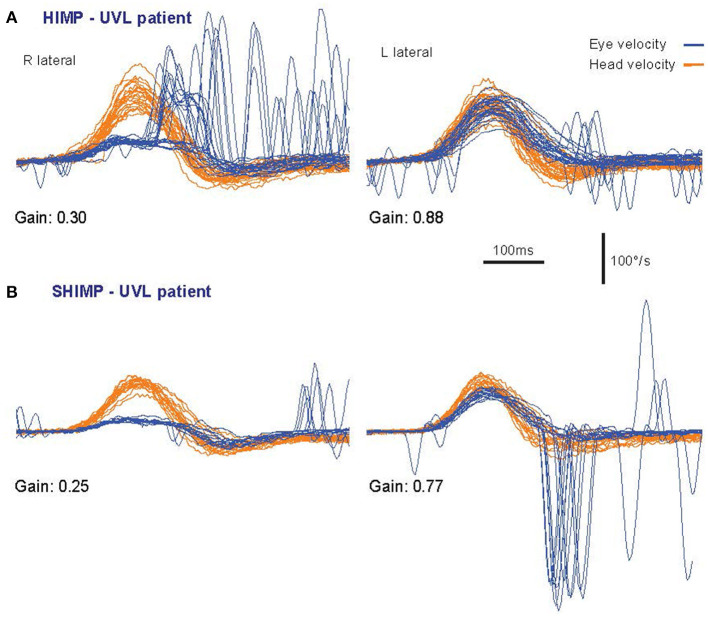
Superimposed time series of the head velocity (orange) and eye velocity (blue—inverted for direct comparison) for repeated individual HIMPs and SHIMPs head impulses. The data are for the same patient with a right unilateral vestibular loss (UVL) during head turns to the affected (R) and healthy (L) side. For head turns to the affected side in both HIMPs and SHIMPS the patient's eye velocity is less than head velocity, and so VOR gain is reduced. In **(A)** HIMPs there are covert and overt corrective saccades at the end of the head impulse. In **(B)** SHIMPs the same patient has no covert saccades during the head impulse to the affected side. For head turn to the healthy side there are many saccades at the end of the impulse (in an anticompensatory direction). Changing the instructions has little effect on VOR but dramatic effects on saccades [reproduced from Curthoys and Manzari (11) with permission].

The 1988 report showed that other oculomotor control systems (optokinetic, cervico-ocular, pursuit) were not able to generate such a fast slow phase compensatory eye movement response within the first 100 ms (2). In the HIMPs test inadequate compensatory SPV is corrected by saccadic eye movements. Saccades occur because the subject is instructed to maintain gaze on an earth-fixed fixation target, and if that fixation is lost, to regain it quickly. Corrective saccades are not generated directly by semicircular canal stimulation, as shown by the fact that delivering similar head impulses but changing the instructions, keeps VOR gain constant but completely changes the pattern of saccades (see Figure 2). Initially the adequacy of the eye movement response was measured by the ratio of the eye velocity to head velocity during the impulse and was referred to as VOR gain, although more recently we have used a position gain which is explained below.

The development of the goggles and the software for vHIT took years (1996–2003) because it required a high-speed camera which could be mounted simply on a pair of tightly-fitting lightweight goggles with minimal slip, and a lightweight head velocity sensor which would give accurate information about the head velocity in the plane of the stimulated canals. The configuration of the goggles on the head is important as we show below. vHIT seems to be such a simple test that clinicians might be tempted to use it without fully understanding how the response depends on the configuration of the goggles and the canals and the trajectory of the head impulse for vertical canal stimulation. In this review, we bring these matters to the fore since they are important in understanding patient vHIT responses.

The subjective “clinical sign of canal paresis” was replaced in 2009 when we published a report of the video system which the Sydney team had developed and validated for actually measuring the eye and head movement during the head impulse—vHIT (10). This was followed in 2013 by vHIT for vertical canal testing, again with scleral search coil data to validate the method (9, 12). In the course of this work, much of the effort was devoted to developing accurate methods for using the two-dimensional (horizontal and vertical) video image to measure the three-dimensional eye movements of patients. Based on those papers, Otometrics (Taastrup, Denmark) developed the ICS Impulse goggles and Halmagyi, Curthoys, MacDougall, and McGarvie acted as unpaid consultants to Otometrics. This arrangement was terminated in 2018. In this review we will focus on ICS technology, but the main principles apply to all the many vHIT systems which are now available.

In the head impulse test inadequate slow phase eye velocity is corrected for by saccadic eye movements. This led us to develop a complementary test paradigm where the patient is required to fixate a *head-fixed* target (a spot projected on the wall from a head-mounted laser) during the same head impulse (Figures 1B, 2). We called this test paradigm SHIMPs (for suppression head impulses) (13). The SPV during the first 100 ms of the eye movement response in SHIMPs is almost identical to that during HIMPs, but the corrective saccade pattern is totally different: in SHIMPs healthy subjects make overt saccades at the end of the impulse because they do not suppress their VOR during the first 100 ms (14). The result is that in healthy subjects the VOR drives the gaze off the head-fixed target during the head turn and so the subject must make a corrective saccade at the end of the impulse. The SHIMPs paradigm shows the dissociation between SPV and saccades which we discuss further below. SHIMPs is an excellent tool for desaccading the eye velocity record in patients with vestibular hypofunction which allows calculation of VOR gain with greater accuracy. But as is clear HIMPs and SHIMPs are complementary ways of testing canal function. These matters are reviewed in (1, 15, 16).

Clinicians have raised questions about some aspects of the vHIT responses they observe (e.g., the importance of head and goggles position, the importance of gaze direction for testing vertical canals, the relationship between VOR gain and saccades), and part of this review seeks to answer some of those questions using published anatomical, physiological, and behavioral data. We caution that vHIT recording systems need to be supported by rigorous published validation evidence in order to provide acceptable accurate data. It is important to use vHIT for the test conditions that have been validated. As will be shown below some apparently minor changes in testing procedure are being carried out without due recognition of the effect of modification on the geometry and physiology underlying the test. For example changing head pitch tilt changes the orientation of the head velocity sensor, as we explain below, and so will change VOR gain.

## Pathways

There are fast pathways from receptors in the semicircular canals *via* the vestibular nuclei in the brainstem to the eye muscles to allow for the very short latency (~7 ms) of the VOR response (17–21). So, each head impulse is giving information about both (1) the functional state of the canal receptors and (2) neural pathways generating the slow phase eye velocity response, but also the functional status of the pathways responsible for generating the corrective saccadic eye movement responses. The neural evidence shows that both SPV and saccades are affected by both cerebellar and/or brainstem dysfunction (22). This review will focus on peripheral mechanisms, fully acknowledging that the VOR is mediated and controlled by central pathways whose status can determine the characteristics of the response. For example, cerebellar deficits or deficits of the medial vestibular nuclei can affect the VHIT response—increasing or decreasing the VOR gain (23). Recently these central aspects have attracted particular attention because vHIT provides a functional test of vestibulo-ocular pathways. The challenge is to understand the changes in vHIT responses due to deficits in the semicircular canal or in structures which influence the transmission of information along the pathways.

“Quality control tests” conducted before the vHIT test—“screening tests”—act to ensure the patient has normal cerebellar function and normal oculomotor range. One way of getting some guidance about the integrity of central pathways is either by examining the patient's smooth pursuit of a slowly moving horizontal visual stimulus, or by giving the patient low frequency sinusoidal horizontal head turns (at about 0.2 Hz) while they fixate an earth fixed target. This tests the visual-vestibulo-ocular reflex (the VVOR). Healthy patients should be able to do this with minimal saccadic involvement. These pretests should be the first step of vHIT testing.

## Assumptions

A huge number of papers have been published reporting the results of HIMPs and SHIMPs because vHIT is such a very simple, fast, innocuous clinical test. Behind the superficial simplicity of vHIT are many major assumptions and factors and they should be recognized. The importance of some factors (such as head pitch position, and gaze direction during vertical canal testing) are especially important and are discussed in detail below [from (24)].

In order to use a head mounted camera to measure the eye movement response during high acceleration head turns it was necessary to analyze the geometry and validate that analysis and assess its significance. We think it is important to clarify some of these matters and address them now to improve the quality of vHIT testing since these matters affect the measured slow phase eye velocity.

In the 2009 paper we did not discuss in detail a number of assumptions which we had analyzed and published (25, 26). Some of the issues which had to be addressed were:

1. The image of the spherical surface of the eye is projected onto the flat surface of the camera sensor so geometrical correction was needed to ensure accurate measurement of eye rotation (25–28).
2. The detailed geometry upon which vHIT is built makes assumptions about the axis of eye rotation and the axis of head rotation (25). In healthy subjects, with all 10 peripheral sensors working correctly, the axes of head rotation and eye rotation will be closely parallel as shown in (29). However, when one or more of the contributing vestibular sensors experiences a functional deficit, the axis of eye rotation may no longer be parallel with the axis of head rotation, changing over the period of the impulse [(29) and Figure 2 of (30)] . This change has important implications for eye velocity measurement as explained below.
3. The software measures the location of the center of the pupil; however the visual axis may not precisely align with the pupil center. Usually this is of no importance, but it may be the source of some microsaccades (see below).

## Geometrical foundations of vHIT testing

At face value, vHIT seems to be a very simple test, however, the one-dimensional quantitative output is actually due to a combination of several three-dimensional systems interacting. These include:

- the orientation of the head in space
- the three-dimensional anatomical combination of the six semicircular canals and their orientation within the skull and with respect to each other
- the three-dimensional components of the impulse delivered to the head
- the location and orientation of the head velocity sensors in the goggles in relation to head position
- the rotation and translation of the measured eye in space with respect to the target
- and the orientation of eye gaze within the orbit.

The following section examines how these factors influence the output for eye and head velocity—and thus VOR gain—which are measured by the vHIT device. As soon as one factor is changed the whole output is affected—not due to a change in the VOR, but due to different measurement conditions. The results of the ICS Impulse device are only validated for certain constellations of the three-dimensional systems mentioned above, which will be explained in the following text.

### Measurement of eye velocity

**Figure 3:**
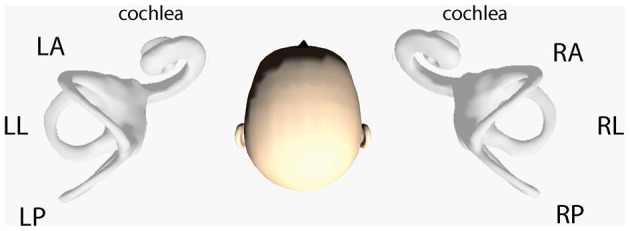
Schematic representation of the orientation of the semicircular canals in the human head. There are three pairs of canals—left lateral (LL) and right lateral (RL), left anterior (LA) and right posterior (RP), and right anterior (RA) and left posterior (LP). The vertical canal pairs are referred to as RALP and LARP in the text. The canal planes are generally not mutually orthogonal and their orientation in the skull varies between individuals as we discuss below (Reproduced from iPhone app aVOR).

The vHIT test, at its most basic, reduces the complex three-dimensional eye movement response to a one-dimensional output. The camera sensor detects the horizontal and vertical position of the center of the pupil, whereas the eye can execute horizontal, vertical, and torsional responses to head movement stimuli. The software differentiates the eye position to eye velocity, but the camera sensor does not detect torsional eye responses. For the usual horizontal HIMP tests, the measurement concerns the relation between the horizontal eye velocity to the horizontal head movement. Vertical eye movement components are also present, although usually small. The numerical output of the eye movement responses in a vHIT test is a single component of the rotational eye velocity in either the horizontal or the vertical plane, and it is compared to the corresponding single component of the head velocity. For testing of the vertical canals in the LARP and RALP planes (see Figure 3), the situation is more complex and will be discussed in detail below. It should be noted that, regardless of the measurement technique involved (coils or vHIT), there is currently no effective technique for separating out the effect of lateral head or eye movement (translation) overlaid on top of the rotational response.

### Measurement of head velocity

#### Theoretical background: Sensor vs. stimulus

**Figure 4:**
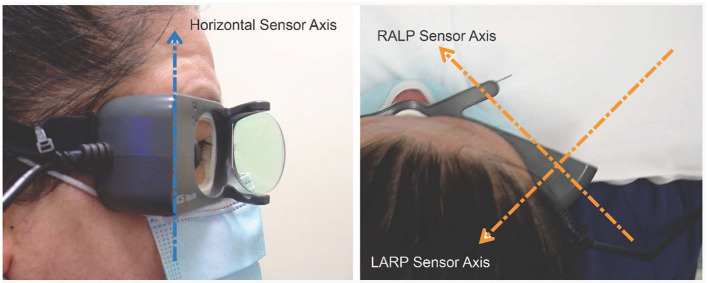
Orientation of the velocity sensor axes in the ICS goggles: Each one-dimensional head velocity output is the signal transduced around that axis by the velocity sensor. Arrowhead gives positive axis direction (right-hand rule) (with consent of person imaged).

Another crucial factor is how the head movement is actually sensed and measured. The orientation of the head velocity sensors in the video goggles is significant for the calculation of VOR gain. For the ICS Impulse goggles, the inertial sensors are set to align with the three stimulation planes of the test. These are the horizontal, the LARP and the RALP planes, with the axes shown in Figure 4. Note that if the goggles are tightened onto a very small head—such as for testing young children—the angular orientation of the sensors changes which affects the head movement data from the goggles, and the measured VOR gain will be affected.

**Figure 5:**
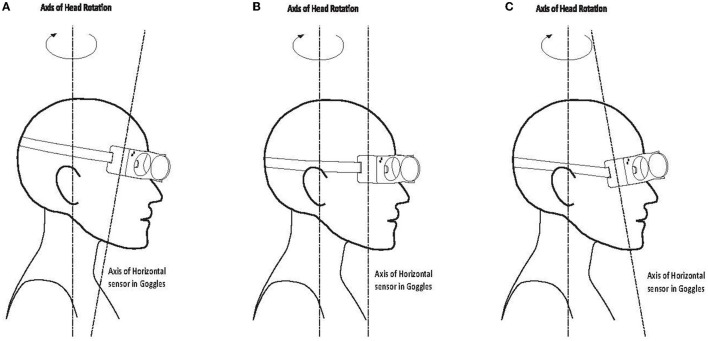
Misalignment between head stimulus axis and horizontal sensor axis of the goggles: **(A)** 10 deg downwards/forward tilt; **(B)** axes aligned; **(C)** 10 deg upwards/backward tilt.

The horizontal head velocity sensor stimulation axis is set to be perpendicular to the horizontal plane of the goggles. Therefore, if there is a misalignment angle between the “earth-horizontal” head stimulus plane and the horizontal plane of the goggles, the measured horizontal stimulus will be reduced by the cosine of the angle between the axes. This means that there are two primary factors which can affect the measurement of horizontal head velocity. The first is the angle at which the goggles actually sit on the face of the subject. Generally, this factor will be minor, with the angle of tilt typically being in the range of plus or minus 10°, which will only lead to a reduction in horizontal head velocity measurement of 1.5%, as shown in Figure 5.

**Figure 6:**
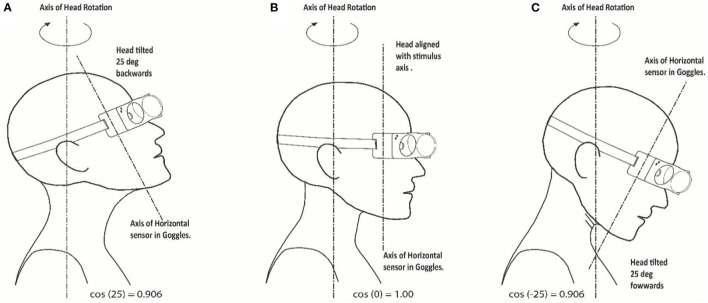
Misalignment between head stimulus axis and tilted head/goggles axis: sketches indicating the axis orientations. **(A)** 25 deg upwards and backwards; **(B)** stimulus and head axes aligned; **(C)** 25 deg downwards and forward tilt.

The second and potentially more important factor is the angle of head tilt during the delivery of the head impulse. The test has been developed for the head to be upright during the stimulus, so that the horizontal head velocity stimulus is correctly aligned with the horizontal sensor. If the head is tilted forward or backward during the test with respect to the stimulus axis, the projection of the delivered “earth horizontal” motion onto the sensor will be reduced by the cosine of the combined misalignment angle of head tilt and goggles tilt with respect to the plane of stimulus. This is shown in sketch form in Figure 6 for a head tilt of 25°. This can be a significant factor if the angle of misalignment between the stimulus axis and the axis of the horizontal sensor in the goggles is large, leading to an apparent increase in the measured VOR gain which is due to the reduction of the measured head velocity (scaled by the cosine of the angle between the axes), while the measured horizontal eye velocity is not similarly affected. Thus, the gain calculated from the ratio of eye velocity divided by head velocity will increase as the measured output of head velocity decreases with increasing angle. At the 25° tilt shown in Figure 6, the measurement of the horizontal component of the head velocity will be cos (25°) or 0.906 of the true value.

### 3D orientation of the six semicircular canals

#### Implications for stimulation in the earth-horizontal plane

The next essential point concerns the 3D orientation of the semicircular canals within the skull and with respect to each other. Studies have confirmed that the horizontal semicircular canals usually lie in a plane tilted roughly 20°-25° above Reid's line (4) (the line connecting the infraorbital margin with the upper margin of the external auditory meatus), which is itself tilted upwards by about 7° from the earth-horizontal plane when the head is upright. Testing of the horizontal canals with ICS goggles has been validated for the head in the earth-horizontal plane with the gaze straight ahead and the goggles square on the face.

However, it has been suggested (31) and tested (32) that the sensitivity of vHIT could be increased if the head was pitched forward to bring the horizontal canals into the plane of the rotation stimulus. However, that suggestion does not take into account the orientation of the head velocity sensor within the goggles (see above), and the neural input from the vertical canals. While it is generally considered that the canals are set roughly orthogonal to each other, the anatomical evidence from (6) and recent anatomical work from (5) shows that this is a simplification.

**Figure 7:**
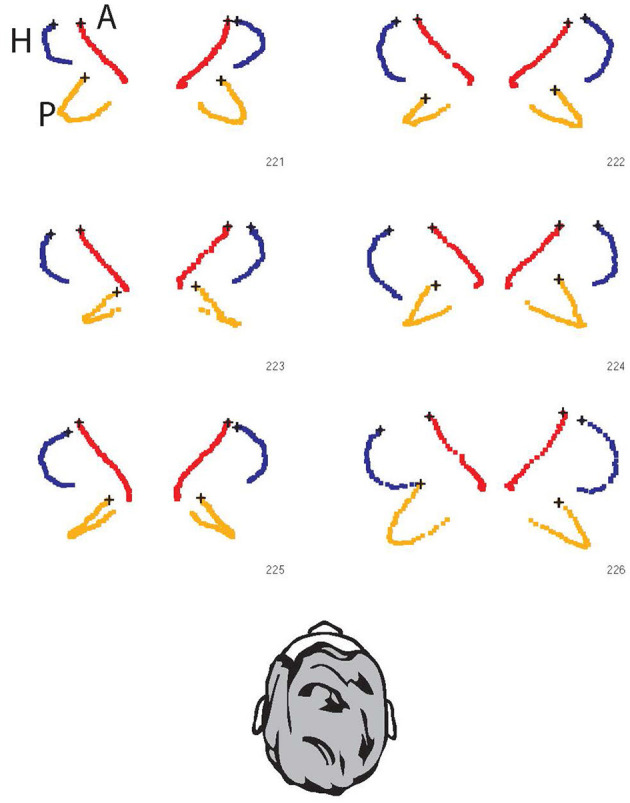
The projection of all six semicircular canals onto the horizontal stimulus plane for six individual skulls. The points are the raw data of points along the bony semicircular canals of six skulls [data replotted from (6) with permission]. Blue: lateral canals; Red: anterior canals; Orange: posterior canals. In all cases, the ampulla is indicated by the black x. The response of the canal to rotation on the horizontal plane can be assessed visually by the area encompassed by the projection of each canal. So in each case the horizontal canals have the largest projected area, and in each case the anterior canals have minimal projected area and so minimal stimulation. However, it is the posterior canals which show large projected areas (e.g., 221 and 226) with large differences between individuals—e.g., in 223 and 225 both posterior canals have minimal projection into the horizontal plane, but in 221 and 226 the orientation of the posterior canal in the head is so large that it has a sizeable projection into the horizontal plane, and so will be stimulated during a horizontal head turn (see also Supplementary Figure 2). These projections correspond to those in Figure 5C of (5) and may appear small, but recall the angular acceleration in vHIT testing is very large (of the order of 2,000–3,000 deg/s/s) so that even a small areal projection will result in effective stimulation of the canal.

However, if the head is tilted forward to bring the horizontal canal into the plane of stimulus as has been suggested, then the anterior canals will now project onto the plane of the stimulus and will contribute to the response, while the contribution from the posterior canals will be reduced as they are brought into a more upright orientation. An excitatory stimulus of a horizontal canal (left rotation for left horizontal canal) is the result of ampullopetal endolymph flow, while in the vertical canals the excitatory stimulus is ampullofugal endolymph flow (pitch forward rotation for anterior canals and pitch backwards for posterior canals). In Figure 7 the ampulla location is marked by the black X. For the posterior canals, the ampulla is at the lower or inferior end of the canal. This leads to an interesting consequence. If there is an appreciable projection of the posterior canals onto the horizontal plane, then a head rotation to the left will excite the left horizontal canal and also excite the right posterior canal by the proportion of the projection of that canal into the horizontal plane. Simultaneously, the right horizontal and the left posterior canals will be inhibited. Inputs from all four canals will generate the “horizontal” VOR response. Thus, individual variants in the 3D configuration of the semicircular canals contribute to the inter-individual differences in the measured VOR response (see also Supplementary Figure 2).

### Experimental validation

**Figure 8:**
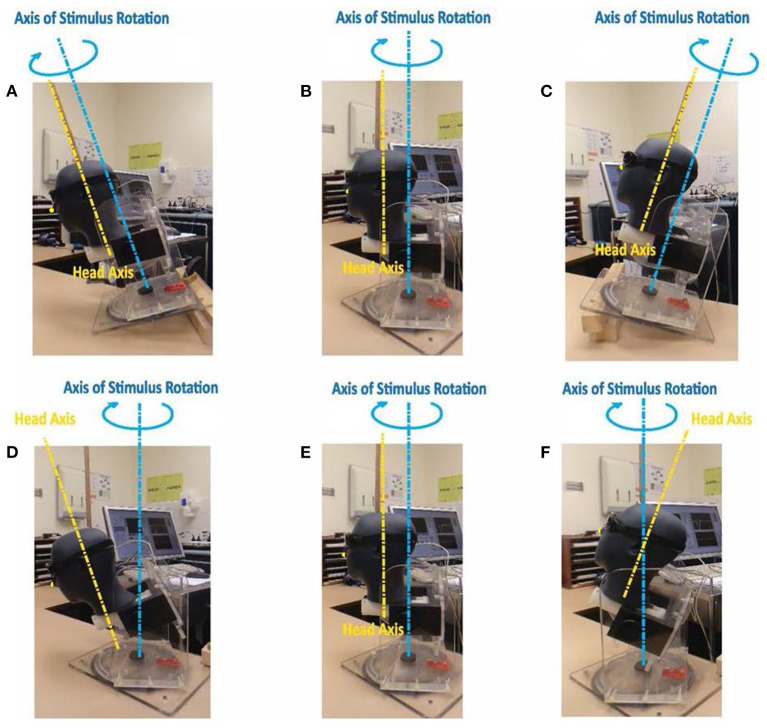
Test jig for determining 3-D goggles outputs during head rotations around axes as shown. Upper row: head and jig stimulus axes aligned: **(A)** jig and head tilted 25° forward; **(B)** jig and head upright; **(C)** jig and head tilted 25° backwards. Lower row **(D–F)** jig stimulus axis earth horizontal: **(D)** head axis tilted 25° forward; **(E)** head axis upright; **(F)** head axis tilted 25° backwards. For impact on head velocity sensor output see Figure 9 (the nose is not clear in these figures and so we have added a small yellow dot on the nose to identify it).

In order to ensure that true earth horizontal impulses were being delivered to the tilted head, the required parameters were determined by use of a model head mounted in a calibration jig normally used to calibrate scleral search coils within a magnetic coil field. Goggles were mounted on the model head and the three-dimensional sensor outputs were determined for upright, earth-horizontal/head tilted, and head axis/ head tilted impulses as illustrated in Figure 8.

**Figure 9:**
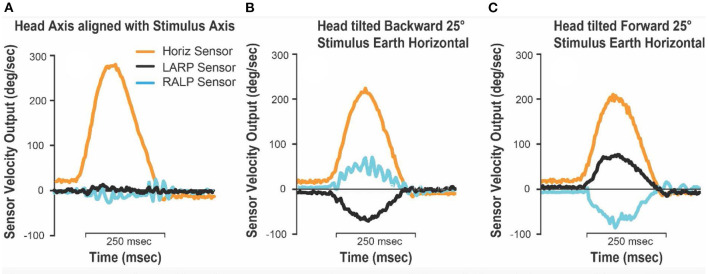
Goggles 3-D head sensor outputs for test jig leftwards earth-horizontal impulses. **(A)** Stimulus axis and head axis aligned (upright, forwards and backwards); **(B)** head tilted 25° backward, jig impulse earth horizontal; **(C)** head tilted 25° forwards, jig impulse earth horizontal. Note that when the head is tilted forward or backwards, and the stimuli are delivered in the earth horizontal plane, the horizontal head velocity sensor underestimates the true horizontal head rotation velocity by the cosine of the misalignment angle. In this case it will be underestimated by about 10% (cos 25° = 0.906).

In all cases where the stimulus axis was aligned with the head axis there were negligible signals from the LARP and RALP sensors (Figures 8A–C, E), and the impulse was correctly sensed by the horizontal sensor in the goggles. An example of the head sensor outputs for those situations is presented in Figure 9A. However, when the head was tilted forward or backwards while the stimulus was delivered around an earth-horizontal axis (Figures 8D, F), the horizontal signal was reduced by the cosine of the angle while the LARP and RALP sensors detected equal and opposing signals due to the sine of the misalignment angle. Figure 9 shows the signals from the goggle axis sensors for leftward earth horizontal impulses, with panel B having the head tilted back 25° while panel C has the head tilted forward 25°. These measurements confirm the theoretical matters presented above—and confirm a horizontal head impulse is a predominant, but not 100% specific stimulus to the horizontal canals as previously noted (6).

### Implications for stimulation in RALP and LARP planes

To test vertical canal function it is necessary to deliver the stimulus in the plane of the tested canals, in other words: the left anterior-right posterior (LARP) or the right anterior-left posterior (RALP) because these canal pairs constitute a functional pair just as the two horizontal canals constitute a functional pair. There is commissural inhibitory interaction between these vertical canals, so the proper test of vertical canals requires that the impulse be delivered in the plane of the canal pair. The stimulus of vertical canal testing is measured by sensors within the goggles which are aligned (approximately) with the plane of the vertical canals—Left Anterior Right Posterior (LARP) and Right Anterior Left Posterior (RALP) (see Figure 4), so the goggles should be configured and oriented correctly on the head for accurate VOR measurements.

### Gaze orientation within the orbit

This factor is of utmost importance for measuring the VOR of the vertical canals, as all four vertical canals can potentially contribute to the SPV eye response, with the relative contribution of each canal depending on gaze direction. It is vital to have the most accurate measures of the eye movement response in order to identify vertical canal function. Using existing technology this is not easy—the reason being that stimulation of a single vertical semicircular canal causes an eye movement response which has both vertical and also torsional components (33, 34). Present video technology is, to the best of our knowledge, not capable of high-speed measurement of ocular torsion—it is restricted to measuring the horizontal and vertical components of the eye movement response at high speed. The torsional component is not measured. To overcome this deficit, we used the fact, shown by 3-D scleral search coil recordings, that when gaze is directed along the plane of the stimulated vertical canal, the torsional component of the eye movement response is minimized (35). However, it is difficult to obtain good results for vertical canal testing, in part because the eyelids can obscure the pupil and so it is necessary to verify that the camera has a clear unimpeded image of the pupil for the full angular extent of the vertical head impulse.

### Implications for “perfect” vertical canal stimuli

**Figure 10:**
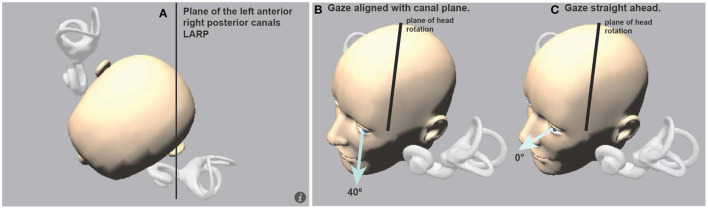
**(A)** View looking down on a schematic head with enlarged semicircular canals to show the approximate planes of the vertical canals. **(B)** The optimal horizontal eye position for using vHIT to measure vertical canal function: it is gaze aligned with the canal plane under test, in this case, LARP stimuli are delivered and gaze should be along the LARP plane. **(C)** If gaze is straight ahead (0°), the prediction is that the vertical eye velocity component becomes much smaller (Figure 11) [Reproduced from McGarvie et al. (36) with permission].

**Figure 11:**
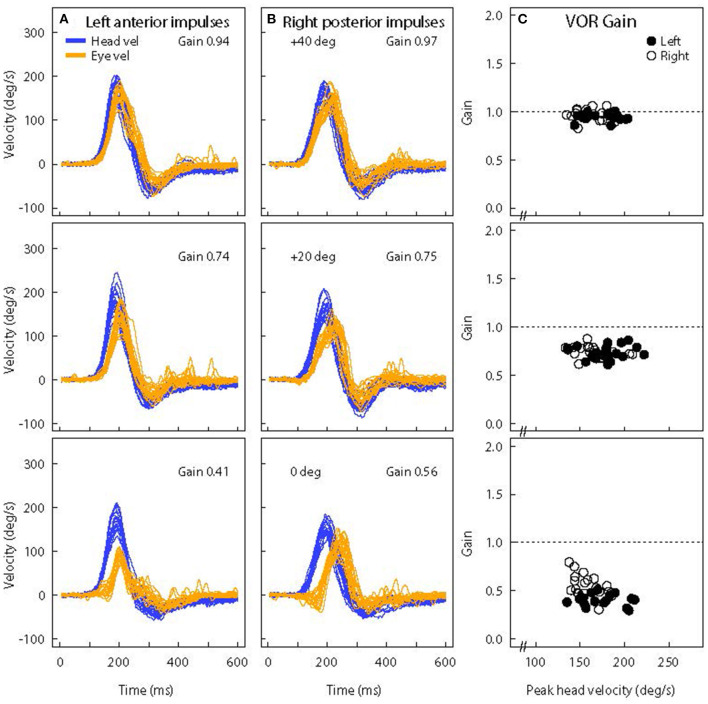
Superimposed time series of eye and head velocity for a number of LARP head impulses of the same healthy subject at three horizontal gaze angles-Aligned with the LARP canal plane (40°) (top row) and at 20° (middle row) and 0° (bottom row). The usual convention is followed-Eye velocity has been inverted to show how closely it matches head velocity. As horizontal gaze moves away from the canal LARP plane **(A, B)**, the measured vertical eye velocity decreases and so the VOR gain decreases **(C)** and at 0° it appears that the whole eye velocity response is delayed [Reproduced from McGarvie et al. (36) with permission]. The head velocity stimulus is the same in each case, but just changing gaze position reduces the measured VOR gain from around 1.0 to around 0.5. However, note that the reduced VOR gain is not corroborated by corrective saccades. The lack of corrective saccades in vertical canal testing with reduced VOR gain, is an indicator that it is incorrect gaze direction which is causing the apparent reduced VOR gain.

In clinical testing with the ICS Impulse version of vHIT, this issue of torsional contribution has been solved by stimulating the patient by abrupt head impulses in the LARP and RALP planes whilst requiring the patient's gaze to be directed along the plane of the canal pair being stimulated (Figure 10). The direction of gaze during vertical canal impulses is critical. When gaze is along the plane of the canal pair, torsion is minimized and the eye movement response to a LARP or RALP head impulse is essentially vertical in healthy subjects. So, a vertical head impulse in the plane of the tested canals is measured as a vertical head velocity, and it elicits a vertical eye movement response with the VOR gain close to 1.0 (9). Inadequate semicircular canal function is shown by smaller VOR gain together with confirmatory corrective vertical saccades. Most importantly, if the results show that the VOR gain is reduced, but there are no vertical corrective saccades, that usually signifies that the person's gaze has not been appropriately directed along the plane of the vertical canals under test (Figure 11).

Figure 11 shows the vertical head velocity and eye velocity raw data for one typical healthy subject with similar head velocity stimulation but with three gaze positions: 40°, 20°, and 0°. When gaze is in the plane of the canal pair being evaluated the gain is in the normal range (0.94). As gaze is directed horizontally away from alignment with the canal plane, the peak vertical eye velocity declines and so the measured LARP VOR gain decreases (36). Notice that the form of the eye velocity time series changes as gaze is moved horizontally away from LARP canal plane alignment—the peak eye velocity response decreases and appears to have a delay relative to head velocity. This kind of pattern of an apparently “delayed” eye velocity response is an indication that the horizontal gaze position is not aligned with the plane of the vertical canals under test. It is due to the fact that when gaze is straight ahead the initial eye movement response is ocular torsion. But as gaze aligns with the plane of the canal, that torsion movement becomes vertical eye movement.

Notice also that at extreme gaze angles (e.g., 0°–straight ahead) the eye velocity is very small, and so VOR gain is very small, although the head movement stimulus is just the same as for 40°. One indicator that this VOR gain measure at straight ahead gaze (0°) is not valid, is that although the measured VOR gain is so small (around 0.5), there are no covert or overt saccades to corroborate that apparent peripheral loss of semicircular canal function. This is an example of how the confirmatory nature of saccades is so valuable in testing. Here, the apparently reduced vertical VOR gain at 0° is a consequence of the oculomotor kinematics—the torsional component is large, so the total eye speed matches head speed, and so there is no need for any corrective saccade.

The “perfect” vertical canal stimuli described above are however hard to achieve in clinical testing. In the Supplementary material, we show that “imperfect” stimuli with pitch and roll components influence the VOR response depending on gaze direction (see Supplementary Figure 1).

### Summary

- vHIT tests should be carried out with the goggles square on the head, the head upright and, for the horizontal impulses, the impulse stimulus axis orthogonal to the earth-horizontal plane.
- The target should be set at eye level at a constant distance (ideally 1 meter) from the subject.
- For vertical impulses, the axes of head movement should align with the LARP and RALP measurement axes within the goggles, and the gaze must be maintained as close to the stimulus plane (the plane of the canal pair under test) as possible.

## VOR gain

### Different methods of VOR gain calculation

**Figure 12:**
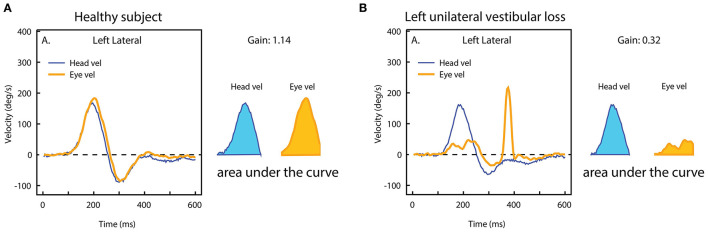
The area VOR gain. The area under the eye velocity during the head impulse is divided by the area under the head velocity. The ratio of the areas in **(A)** is around 1.0, but in case of unilateral vestibular loss **(B)**, the eye velocity area is much smaller, and so the area VOR gain is much smaller (0.3) [Reproduced from Curthoys and Manzari (11) with permission].

One continuing issue in vHIT testing is VOR gain. The demand from most people carrying out the tests is to reduce this complex VOR response to a single number. Historically, the two main techniques to quantify VOR performance have been velocity gain (also called instantaneous gain), i.e., the ratio between eye and head velocity at a given point in time, and regression gain, which gives the ratio between the slopes of eye and head velocity over a given period (37). However, (9) determined that a position gain obtained by dividing the area under the desaccaded eye velocity curve by the area under the head velocity curve over the duration of the impulse, was the most effective technique to minimize the quantitative effect of goggles slip artifacts on the vHIT. The position gain (also called the area gain) is calculated from the onset of the impulse until the moment the head velocity once again crossed the zero line (see Figure 12).

In early studies of the VOR, gain was defined using engineering technology—gain was measured by taking the ratio of eye velocity to head velocity during maintained sinusoidal horizontal angular acceleration. But for isolated single stimuli, such as individual head impulses, this has problems because the ratio of eye velocity to head velocity changes during the head velocity stimulus. The ratio at a single point is especially unreliable at small values of head velocity where measurement error can dominate any gain calculation. So when we initially reported the detailed quantitative data about eye velocity during head impulses using scleral search coils (3) we solved that problem by reporting VOR gain as the ratio of eye velocity to head velocity measured at one very high arbitrary head velocity (122.5 deg/s). This high velocity was chosen because it is well-away from the changes which occur at the start of the head impulse when both eye and head velocity are small and thus prone to errors generated by the inaccuracy of the quantitative measure of those two velocities. This is especially the case for vHIT, where goggle slippage at the onset of the head movement may cause errors in head velocity [for details see Figure 9 in (38)].

However, this VOR gain measure at a single point is subject to artifacts and errors because it is such a localized measure at just one moment during the complex eye movement response. Other systems have chosen to use the instantaneous VOR gain at two or three moments during the head impulse. In 2013 we went back to basics by asking: why do patients with a unilateral loss make a corrective saccade? And the answer is that they make a saccade because their gaze at the end of the head impulse is not on target. In other words it is gaze position error, which drives the generation of a corrective saccade. To measure gaze position it is necessary to integrate eye velocity during the entire head impulse and compare that to the integrated head velocity record. VOR gain is then the ratio of these two areas (Figure 12). And that is the way the ICS Impulse system calculates position (area) VOR gain. This calculation is done after saccades are deleted from the eye velocity record during the head impulse using an eye acceleration algorithm to detect the presence of a saccade.

### Absolute gain value

**Figure 13:**
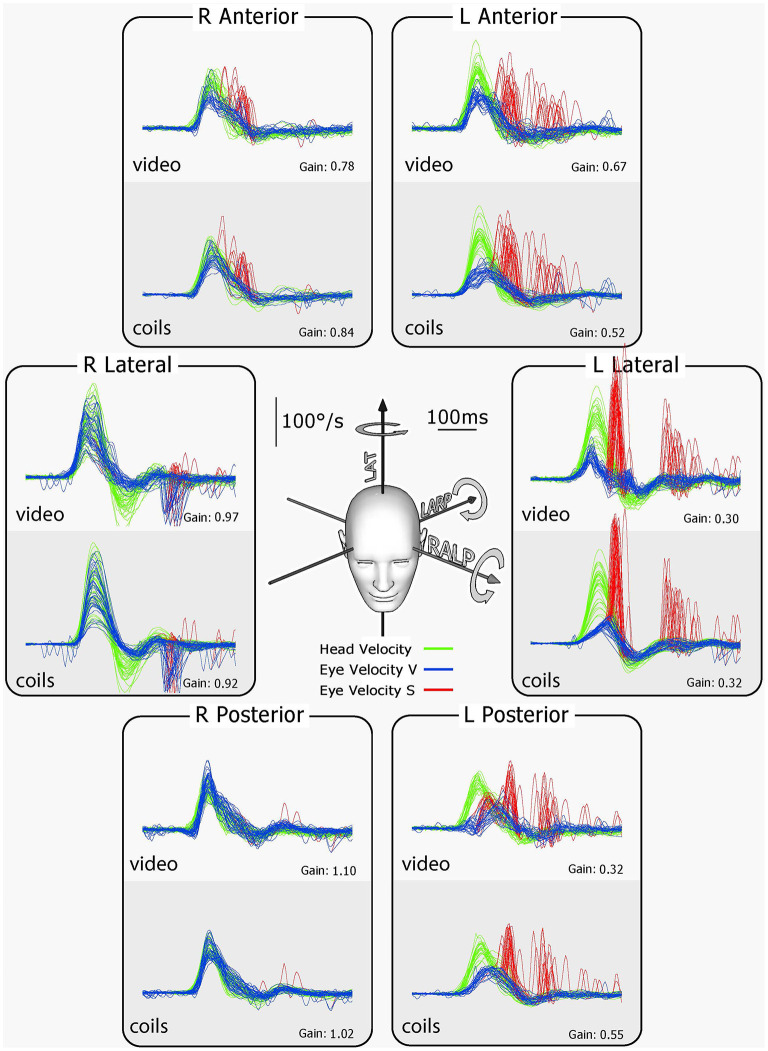
Results for tests of every semicircular canal of a patient with left unilateral vestibular loss. The patient's eye was measured simultaneously with scleral search coils and vHIT. For every rotation direction activating the canals on the healthy right side, the eye velocity response is close to normal. However, for every rotation in a direction to activate canals on the affected left side, there is a reduced VOR response. In particular, for the vertical canals on the affected side, there is clearly reduced function. To compensate for the deficit on the affected left side, overt saccades appear after head rotation (red traces). These corrective saccades are important confirmation that the reduced VOR gain is not due to poor gaze direction. The late inverted overt saccades for right lateral stimulation are likely due to the fact that there was such a big overshoot in head velocity that the overshoot became an impulse to the affected left side, which the affected ear could not generate sufficient SPV to compensate, so the “reverse” overt saccades appeared. There is very close similarity between coils and vHIT traces, further validating vHIT [Reproduced with permission from MacDougall et al. (9)].

vHIT has the notable advantage of providing information about the absolute level of function of each individual canal—in other words it is not simply a measure of the difference between the two sides as is the canal paresis score. This is because for high-acceleration head movement stimuli, any contribution from the opposing canal is effectively eliminated because the high acceleration acts to suppress the neural response of that contralateral canal [see (38) for details]. One should however bear in mind that the VOR response of one particular canal is due to excitation of the ipsilateral canal and—to a much smaller extent—disinhibition of the corresponding contralateral canal (“push-pull” effect) (38, 39). This has two implications for VOR gain in unilateral peripheral vestibular loss. First, in patients with unilateral loss the gain of the affected canal will not drop to 0 (only to ~0.3) because the disinhibition from the healthy contralateral side is still active. Second, the gain of the contralateral canal may also be slightly reduced (0.7–0.8) because the disinhibition from the side with the vestibular loss is missing (see Figure 13).

### Enhanced eye velocity

In testing vHIT, some patients show enhanced eye velocity and so VOR gains >1.0 (40–42). In many cases a VOR gain >1.0 is probably due to incorrect calibration, but we asked the question whether it is possible that changes in fluid dynamics could result in systematically enhanced VOR gain, in particular could enhanced VOR gain be an indicator of endolymph hydrops. The questions become (1) is an enhanced eye velocity a reliable, repeatable observation within a patient and (2) what the mechanism of this enhanced eye velocity may be.

The answer to the first question is yes—VOR gains >1.0 can be reliable within some patients. Jorge Rey-Martinez tested the same patient over years and this patient consistently demonstrated enhanced eye velocity (41). The reliability has also been confirmed in repeated measures on other individual patients (42, 43). The next question becomes: what could be the mechanism for such enhancement? Part of the answer to that originated from studies by Grieser et al. who modeled fluid flow in semicircular canals in patients with semicircular canal dehiscence (SCD) and demonstrated that in fact that there can be enhanced fluid flow in such patients (44). Fluid dynamic modeling has also been applied to explain the results of patients with hydrops by (45) who found that hydrops can also enhance the cupula response to high accelerations (as in vHIT) and so enhance the eye velocity response.

Reliable enhanced eye velocity in vHIT could become an indicator of vestibular hydrops. A sizeable number of patients have demonstrated reliable enhanced eye velocity but given the above analysis of the importance of goggle and head orientation, it is necessary to exclude such factors as the cause of the enhanced eye velocity response. When VOR gains >1.0 occur the usual suggestion is: readjust the goggles, repeat the calibration, and repeat the test to find out if it is a reliable, consistent result. In some cases it is (41).

### Tracking patient recovery

A significant advantage of vHIT is that it can be used to test semicircular canal function repeatedly even at short intervals—even just a few minutes apart. This has been of significant value in tracking the changes in semicircular canal function over time, for example in vestibular neuritis: does a patient recover semicircular canal function, or is it permanently lost? A recent paper reported the results of meticulous monitoring of the VOR by vHIT over 500 days (46). The data was vHIT from each of the six semicircular canals of a single patient with acute vestibular neuritis repeated at many intervals over 500 days, and the results show that spontaneous recovery of semicircular canal function after neuritis can take much longer than generally expected, with consequences for the patient's recovery and rehabilitation.

### Vertical canal VOR gain

In 2013 the evidence about using vHIT to measure vertical canal function was published, again comparing vHIT measures to search coil recordings of the same eye (see Figure 13) (9). The neural pathways for vertical VOR are very different from those for horizontal eye movements, so these results give invaluable additional information not only about vertical canal function, but also about the state of central pathways responsible for the vertical eye movement response, as has been shown for example in patients with internuclear ophthalmoplegia (47). The results have proven how valuable that is—for example the surprising result of bilateral anterior canal sparing in bilateral vestibulopathy due to aminoglycosides and Menière's disease (48) was unknown before vertical canal testing.

## Saccades

The original “clinical sign of canal paresis” was the presence of the saccade correcting for the gaze position error as visually detected by the clinician at the end of a clinical head impulse (2). That corrective saccade is now termed an overt saccade. However, when the eye movement response in the head impulse test was measured either by search coils or more recently by video procedures it became clear that corrective saccades could be produced during the head impulse itself, and since these cannot be detected by visual inspection, these saccades are termed covert saccades (8). Saccade detection then is a most important part of the measurement of the response to the head impulse.

It is eye position which is directly measured by the camera, and eye position is digitally differentiated to yield eye velocity. It is important to realize that relying on eye velocity as the response measure is an excellent way of detecting extremely small but very fast eye movements because even microsaccades (which are less than one degree wide), have very large eye velocities associated with them [e.g., (49, 50)]. This is easily shown by using vHIT to record eye velocity as the person makes a saccade from the left edge of a fixation target (about the size of a 1-euro coin) at 1 meter to the right edge of that target. These tiny saccades may have velocities of 100–150 deg/s.

**Figure 14:**
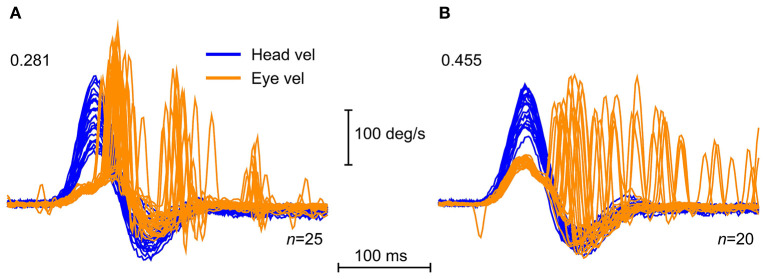
Saccadic clustering. Time series of vHIT for horizontal impulses toward the affected side for two patients after vestibular schwannoma removal, showing examples of clustered saccade pattern **(A)**, and disorganized saccade pattern **(B)**. The clustered saccades here appear in three main time intervals, one toward the end of the impulse, and two more clusters after the impulse. This pattern is characteristic of a patient who is compensating for the loss of function on the affected side. The disorganized saccades of the other patient in **(B)** do not show the same clustering, but are distributed more uniformly after the end of the impulse. The gains of the VOR are shown on each plot [Reproduced with permission from Curthoys and Manzari (11)].

Why do saccades occur? The head impulse test is essentially a gaze position task—the subject or patient is instructed to maintain their gaze on the target during the unpredictable head turn. If semicircular canal function is inadequate, the eye movement will not compensate for the head movement during the impulse and so at the end of the impulse, there will be a gaze position error. The eye will be dragged with the head, off the fixation target so this gaze position error requires a corrective saccade to return gaze to the target. It is our contention that some patients learn to make these saccades during the head impulse. As the patients recover from unilateral vestibular loss, these corrective saccades occur earlier during the impulse and tend to cluster around a particular epoch during the impulse (51, 52). We contend that the change in timing and clustering appears to be the result of a learning process (51). An example is shown in Figure 14.

Some patients with vestibular neuritis tested at the acute stage show a pattern of corrective saccades which are distributed in time so there is no particular time window when the saccades occur—they are not clustered. However, when tested at later occasions, the same patient's slow phase eye velocity (and so VOR gain) has not changed but now the corrective saccades cluster (51, 53). Clustering has been reported to be indicative of improved subjective recovery after vestibular loss (54–57). There is an excellent quantitative measure of clustering called the PR score (58).

One consideration here is that vHIT testing is a learning situation. Patients can learn to change their performance without any apparent feedback, without any explicit reinforcement, so that repeated testing of the one individual may show a systematic change in the occurrence and timing of corrective saccades, whilst their SPV response remains unchanged. Going from the acute stage in which saccades are spread over a wide time range (during and after the end of the head impulse), to responses on later testing where saccades are tightly clustered (covert saccades) during comparable head impulses. We have attributed that change in performance to learning. Because the simple fact is that even though the saccadic pattern may change, the VOR gain does not necessarily change as long as the peripheral vestibular deficit is present (53).

How big can a saccade be before it can be regarded as showing inadequate semicircular canal function? There is no simple answer, because the saccade size and occurrence depend on so many non-vestibular factors: instructions, age, spectacle correction, peak head velocity, all of which can influence saccade size and the timing of occurrence of saccades. In the SHIMPs testing paradigm, patients without vestibular function make no saccades at all because their gaze is not taken off the head-fixed target. There are a host of neural mechanisms which govern saccades. Because saccades are so easy to measure, it is simple to quantify changes in saccadic performance over time and focus on the saccadic characteristics over time and not the SPV. But we stress that saccades are NOT the direct diagnostic indicators of semicircular canal function in vHIT testing, the slow phase eye velocity is the direct indicator.

We are concerned that some clinicians are starting to use saccades as direct indicators of semicircular canal function. That is not acceptable because it is not supported by physiological evidence. Semicircular canals drive the slow phase compensatory eye velocity *via* a short fast three-neuron arc, so it is slow phase eye velocity (and thus VOR gain) which is the direct indicator of semicircular canal function in vHIT testing. Saccades are driven by very different complex neural mechanisms (59).

**Figure 15:**
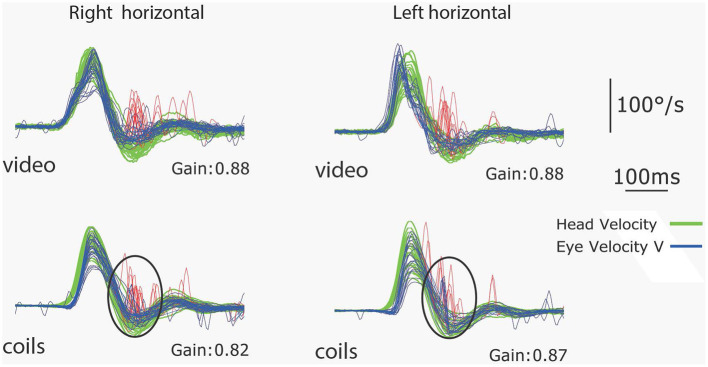
Simultaneous coils and vHIT recordings of the responses of a healthy subject to leftwards and rightwards horizontal head impulses. The VOR gains are in the normal range (>0.8) but this subject makes small saccades (microsaccades) at the end of the impulses which are clear on both video and coils records and are circled on the coils records. This is an example of a subject with normal VOR gain but small corrective saccades [Reproduced from MacDougall et al. (9) with permission].

In our experience many healthy subjects make small corrective saccades (microsaccades) during vHIT testing (see Figure 15).

These corrective saccades have caused some confusion because some authors apparently consider they must be due to pathology, even though the VOR gain is in the normal range. What are the causes and the significance of these very small saccades in healthy subjects with normal semicircular canal function? If a healthy subject's VOR gain is 0.9, [and so within the normal range—[>0.8 but < 1.0] (60)] then at the end of the impulse, their eyes will not be exactly on target, so they will usually make a (very small) corrective saccade to get back to target as instructed (see Figure 15). But as we have noted small saccades—even microsaccades—have high peak velocities and so are very clear on the response record. In fact a healthy subject with a VOR gain of 0.9 may make a corrective saccade because at the end of the impulse their eyes may not be exactly on the center of the target. So saccades with normal VOR gain rather than being an anomalous result in vHIT testing, as some have claimed (61) are in fact a normal response, and our detailed measures with search coils as well as video show that in fact many healthy subjects make such responses.

Other factors affecting saccades apart from SPV, should be considered. If a healthy person with a normal VOR gain tries to keep their gaze to be exactly on the center of the target, at the end of the impulse, they will make corrective saccades even for tiny position errors. As noted above, a saccade from one edge of a fixation target at 1 meter to the other edge of the target may have velocities of 100–150 deg/s. As far as peripheral vestibular function is concerned, these microsaccades are not clinically significant.

### VOR gain + saccades

An index which includes both VOR gain and saccade quantification has been reported to improve diagnostic accuracy (50). This is not surprising because these two indicators are complementary measures, so combining both should improve precision of diagnosis. However, the above analysis shows that saccades do not directly measure semicircular canal function. With the appropriate instructions they complement VOR gain measures, but saccades are subject to many other control mechanisms, so they are not a direct indicator of semicircular canal function. Combining VOR gain and saccades is particularly helpful to follow recovery of the VOR, e.g., after vestibular neuritis (53). Increase of the VOR gain is accompanied by a gradual decrease in corrective saccades because they are no longer required to correct for the position error at the end of the head impulse. This is a very dynamic process which is mainly driven by VOR recovery, but also by many other factors that affect presence and timing of saccades (see below). Within this process, there may be situations where VOR gain has (almost) recovered to normal values, but saccades are still present as an indicator of the original position error (62).

Any process which affects saccades can be reflected in the performance of this simple task. There are a host of mechanisms which affect saccades and because saccades are so easy to measure, it is simple to quantify changes in saccadic performance over time and focus on the saccadic characteristics over time and not the SPV. The dissociation was recently shown by the results of SHIMPs testing in patients with early Parkinson's Disease, who had VOR gain in the normal range but, as expected, significantly delayed saccades (63). It is ironic that our original (Halmagyi and Curthoys) report in 1998 described the clinical sign of canal paresis as the presence of the corrective saccade and this happened because the saccade could be detected relatively easily by clinicians at the bedside. However, what was not stressed then was that the saccade occurred to correct for inadequate slow phase eye velocity driven by inadequate semicircular canal function.

### What can affect saccades?

Foremost is the cognitive aspect- saccades are made in response to instructions! That is most clearly shown by comparing the results of a patient with a unilateral peripheral vestibular deficit, in two versions of head impulse testing: one with the fixation target fixed on the wall (called HIMPs), the other with the fixation target moving with the head (called SHIMPs) (Figure 2). In both cases, the kinematics of the head turn are similar, and the slow phase eye velocity is similar, but the saccadic response is totally different (see Figure 2) (13). Other factors affecting saccades are: age, target size, predictability of head impulse direction and testing in light vs. dark (64–69).

Of course the study of saccades and their interpretation is of very great interest because of all the extra information saccades can provide (66, 67, 70, 71)—e.g., they can be so useful in identifying lesions along the central pathways of the VOR in structures (such as the cerebellum) which modulate the transmission of information along those central pathways (22, 72–75).

## Summary of major issues in vHIT testing—A string of pearls

We have shown that there are a host of variables which can affect vHIT measures, but once the clinician has some experience and practice in carrying out the test, the great value of this fast simple, innocuous test will be apparent. As is clear from this review, attention to detail is vital. In the interests of improving the quality and repeatability of vHIT testing we present the following:

### Goggle position

vHIT tests should be carried out with the goggles square on the head, the head upright and, for the horizontal impulses, the axis of the head turn being perpendicular to the earth-horizontal plane. The target should be set at eye level at a constant distance (at least 1 meter) from the subject. For vertical impulses, the axes of head movement should align with the LARP and RALP measurement axes within the goggles, and the gaze must be maintained as close to the stimulus plane (the plane of the canal pair under test) as possible.

### Instructions

It is necessary that the patients can see the target (some color-blind people have difficulty), and understand the instructions, and that the goggles are tight and have minimal slippage. The participant must be able to identify the target, however it is not required to see it sharply. The test can even be conducted if the person is blind in the right eye but can still see the target with the left eye. The usual instructions to the test subjects are to ask them to maintain fixation on an earth-fixed target at one meter distance because fixation distance affects the actual response [close fixation distance increases VOR gain (76)]. The test can be conducted in full light because visual input in this test has a fairly long latency, so it does not contribute to the early SPV response. Similarly, neck input in most healthy people is very small (77). During the test the clinician needs to keep exhorting the patient to look at the spot, relax their neck and not “help” with three repeated requests:

(1) Watch the target as closely as you can, (2) Keep the eyes open, (3) Let your head be like a rag doll...don't help or hinder...just go with it!

### Repeating the test

Any unusual result requires repeating the calibration and the test. Reposition the goggles, repeat the calibration to find out how reliable this unusual observation really is.

### Head velocity

The peak head velocity of the abrupt passive head turn should be at least 150 deg/s and at least 120 deg/s for the vertical canals, because with smaller peak head velocities patients with inadequate vestibular function can produce eye movement responses which lie within the normal range by using the intact VOR of the contralateral side [see (39) Figure 2].

### Checking the results

First step—inspect the records: “traces before numbers”. Before even considering VOR gain, it is mandatory that the actual eye movement and head movement traces be inspected closely. We even recommend inspecting the head and eye records for each impulse one by one, before examining averaged VOR gain. The reason is that apparently small “glitches” in the individual traces can effectively falsify the clinical test. For example, if the goggle strap is loose, then it may appear that the eye starts to move before the onset of head movements. This is not possible, and the test should be repeated, instead of publishing a “new finding” of vHIT. See also the papers by Mantokoudis on the various artifacts of vHIT testing (38, 78, 79).

### Age

Age-dependent norms for horizontal and vertical vHIT have been published (60) and show little effect of age on VOR gain for either horizontal or vertical canals, with the exception that the VOR gain for the posterior canals is significantly < 1.0 for all ages. For healthy community dwelling people, the VOR is minimally affected by age, at least into their 80s.

### Interocular differences

Our earlier measures with binocular 3D scleral search coils in healthy subjects during a head impulse showed that the two eyes do not move exactly conjugately. Thus, for the leftward impulses, the left eye showed a higher gain than the right eye and vice versa, for rightward head impulses the right eye showed a higher gain than the left eye. In other words: it is the adducting eye which has the higher gain with a side difference of ca. 15% between the two eyes (80). This is probably a cause of some of the sidedness differences which have been reported in the past in carrying out these tests.

### Goggle slippage

It is extremely important that the goggles be protected from any movement artifact. They must be tightly strapped on the head and the operator's hands must be well-away from the goggle's straps. Slippage is the cause of many cases of apparently strange eye movement responses. An eye movement response which starts before the head movement stimulus is due to goggle slippage rather than some kind of occult neurological problem.

### Lid artifact

Check for a clear image during the whole extent of the eye movement with no reflections in the pupil and no eye lid obscuring the pupil. Keep the room well-illuminated—even use a battery driven (DC) light source to keep the pupil small.

### Darkness

Testing with vHIT in complete darkness is not recommended, since darkness results in a very large pupil which is prone to lid and reflection artifacts (see pearl no. 9), thus making accurate measures of eye position very difficult.

### Passive vs. active

The head movement in vHIT is an abrupt, passive unpredictable head turn. If subjects are allowed to execute this movement themselves (i.e., the subject makes an active head turn while gazing at the target) they can use non-vestibular mechanisms to generate a compensatory eye movement response so that—even after vestibular loss—the VOR may superficially appear to be normal (81).

### Number of impulses

Every head impulse is a test of the semicircular canal. In terms of the stimulus, it is equivalent to a caloric test of that canal. Recommendations for conducting 20 impulses in each direction were made to ensure the repeatability of the responses. However, in very difficult or emergency situations, a few impulses are sufficient to give the essential data (82).

### Quality control: Pre-tests

“Screening tests”—i.e., tests conducted before the vHIT test process [e.g., by observing smooth pursuit) or visual-vestibular interaction during slow sinusoidal horizontal head movements (83)]—act to ensure normal central function and a normal range of eye movement. These should be the first step of vHIT testing. Low frequency (0.2 Hz) passive sinusoidal head turns applied by the clinician through the range of head movements ensure that there are no reflections in the pupil or that the pupil is not obscured by the eye lid.

### Quality control: Post-tests

“Traces first, then numbers”. It is absolutely mandatory that the traces be inspected, even one by one, before the VOR gains associated with those traces are studied and reported, for the very simple reason that artifacts and errors can occur which generate incorrect gain values resulting in misdiagnoses. Simply using the VOR gain value without checking the quality and accuracy of the records is unacceptable. For example, a low VOR gain without any corrective saccades strongly points to a testing error or a misunderstanding of the task by the patient.

### Artifacts

Using this head-mounted system allows many artifacts to occur and these have been studied and reported in great detail by (78, 79), the reader is referred to these papers. It is important that people using vHIT understand the basis for hard factual responses.

### The data

Papers reporting vHIT data should include the actual time series records—as thumbnails in Supplementary material.

## Conclusion

vHIT has provided clinicians with a new tool that made it possible for the first time to determine the degree of function of all six semicircular canals. Originally designed to detect peripheral vestibular hypofunction, the test also depicts central vestibular dysfunction and allows insights into the very fundamental basic mechanisms of inner ear operation. Monitoring vestibular function in patients over time has provided new insights into disease processes.

The final conclusion is that the most direct indicator of semicircular canal function using vHIT is the SPV and the VOR gain in its various manifestations. Saccades are not direct indicators of semicircular canal function but are indirect indicators, acting to corroborate or confirm the VOR gain measure and should be recognized as such. Saccades can be influenced by a host of factors apart from a loss or reduction of peripheral vestibular function.

## Author contributions

IC and JD wrote the paper, with help from AB. LM, HM, JR-M, and GH wrote sections. The Section on Geometrical foundations comes from the PhD thesis of LM. HM and LM conducted kinematic analyses to validate the section on geometry. JR-M conducted a simulation to verify canal cross-coupling. All authors revised the paper and approved the final version.

## Dedication

We dedicate this paper to the memory of a brilliant vestibular scholar, scientist and friend, Hans Straka, who died suddenly in December 2022. The understanding of vestibular anatomy, physiology and function which Hans has given us, underpins vestibular clinical testing.
