# Supplementary material for: A review of the geometrical basis and the principles underlying the use and interpretation of the video head impulse test (vHIT) in clinical vestibular testing
Source: Front Neurol. 2023 Apr 11;14:1147253. doi: 10.3389/fneur.2023.1147253 (PMC10126377; doi:10.3389/fneur.2023.1147253)
Supplement: Supplementary file 1 [file Data_Sheet_1.PDF]

Supplementary Material

Implications for "imperfect" vertical canal stimuli

To demonstrate the importance of gaze orientation: as an example, consider a Left Anterior (LA) head impulse stimulus and three different gaze orientations. The three gaze positions considered are gaze oriented in the LARP plane (45 deg), gaze straight ahead (0 deg), and gaze oriented in the RALP plane(- 45 deg). In the case of a normal subject, the axis of eye rotation will be strongly aligned with the axis of head rotation. If the examiner could deliver an absolutely perfect LARP stimulus without any component of either RALP or LA during the
stimulus, the situation would be much simpler (see main text "Implications for "perfect" vertical canal stimuli" and Suppl Fig. 1A). (Suppl Fig. 1A). However, in reality there are always likely to be some additional components of either head pitch or head roll, or even a combination of these two, during a predominantly LA impulse stimulus (Suppl Fig. 1B, D, which includes 20% RALP with 80% LARP; i.e. slightly more head pitch).

As is known from pioneering early work, stimulation of a single canal will drive eye movements around an axis parallel to the axis of that canal (Cohen et al., 1964). Therefore, when gaze is oriented in the LARP plane, stimulation of the left anterior or the right posterior canal will generate a vertical pupil movement, while any stimulation of the right anterior or the left posterior canal will generate a torsion of the eye and no vertical movement of the pupil (Suppl Fig. 1B). Therefore, even with the non-perfect LA impulse, the measured vertical pupil movement will be driven only by the canals oriented in the LARP plane.

Next, consider the situation with gaze oriented directly straight ahead. (0 deg). In this case, stimulation of either set of canals (LARP and RALP) will generate eye movements which have a combination of vertical and torsional components (Suppl Fig. 1C, D). In a pure head pitch forward stimulus, all four canals will combine to drive a purely vertical pupil movement, while in a pure head roll situation all four canals will combine to generate a pure torsion of the eye. If, however, the examiner generates an "imperfect "LA head impulse stimulus (Suppl Fig 1D) then a majority of the vertical pupil movement will be driven by the pitch plane projection component of the vertical LA impulse. However, as the LA impulse is imperfect, there will also be a small vertical component from the pitch plane projection of the RALP canal pair which, depending on how the head is moved, will either add to or subtract from the vertical pupil movement being driven by the LA canal. Consequently, the vertical pupil movement is not purely a response to the left anterior stimulation.

This becomes even more obvious when considering the situation in which gaze is aligned with the RALP plane while the same "imperfect" LA impulse stimulus is delivered. In this situation, stimulation in the LARP plane alone would generate a torsional eye movement and no vertical pupil movement at all. However, since the LA impulse stimulus is imperfect there will be some stimulation in the RALP plane which will now generate a perfectly vertical movement of the pupil. Consequently, with gaze in the RALP plane, any measured vertical pupil motion will only reflect the imperfection of the LA stimulus and will not contain any information about the LA canal function and drive at all. Consequently, in order to accurately measure the vertical canal rotational responses, it is always necessary to ensure that the gaze is correctly aligned with the plane of stimulus.

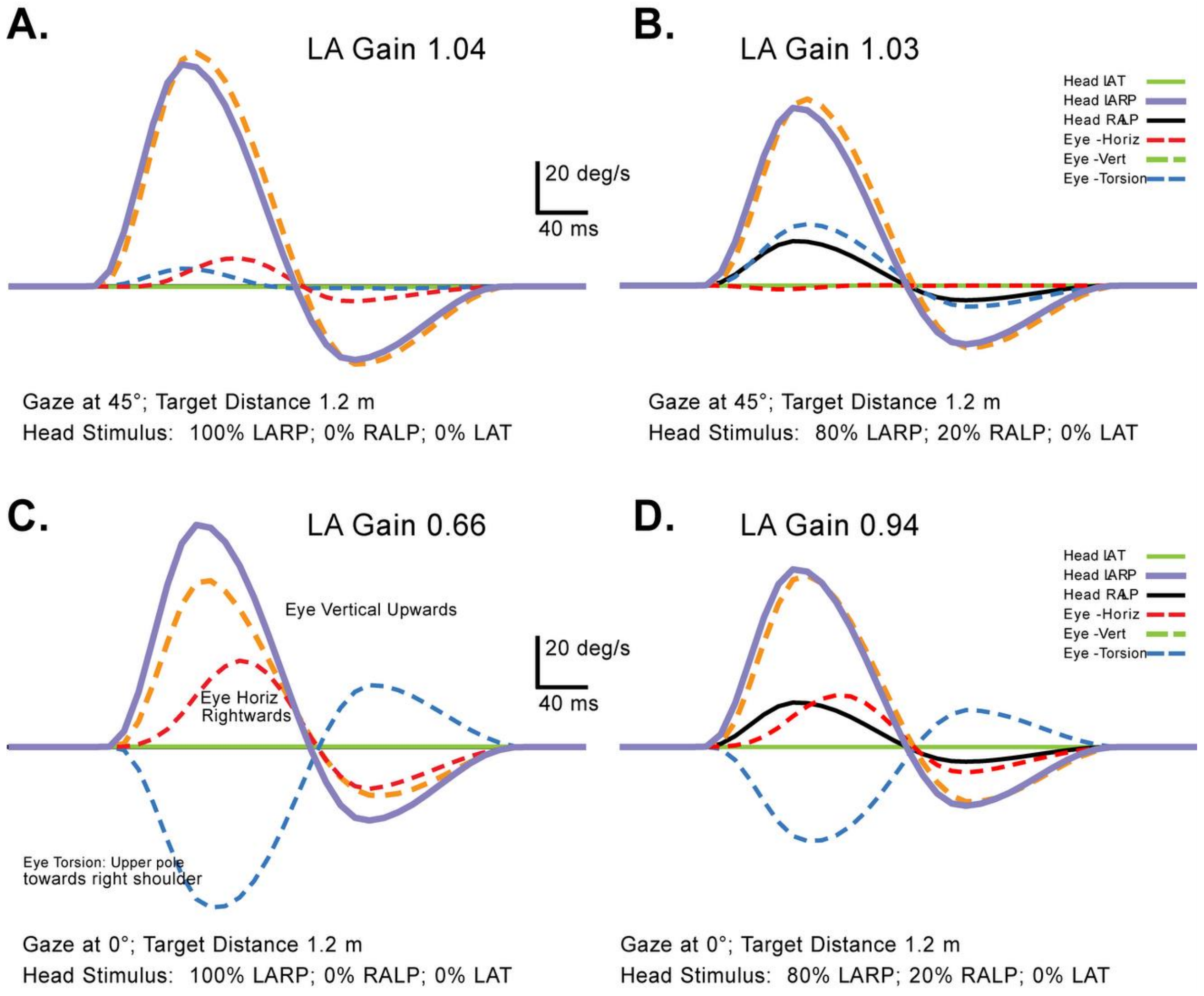

Supplementary Figure 1. Output of inverse kinematics model showing eye rotations required to maintain gaze perfectly on the target during the given impulse (developed by H.M.). Left Anterior Gain calculated by area under vertical Eye velocity divided by area under LARP head velocity A. “Perfect” LA impulse delivered only in the LARP plane; Gaze at 45 deg in LARP plane; LA gain 1.04 B. “Imperfect “ LA impulse delivered with 20% RALP stimulus ,i.e. a bit more pitch; Gaze at 45 deg in LARP plane; LA gain 1.03. C. LA impulse delivered only in the LARP plane; Gaze at 0 deg, straight ahead; LA gain 0.66. D. Imperfect LA impulse delivered with 20% RALP stimulus i.e. a bit more pitch; Gaze at 0 deg, straight ahead; LA gain 0.94.

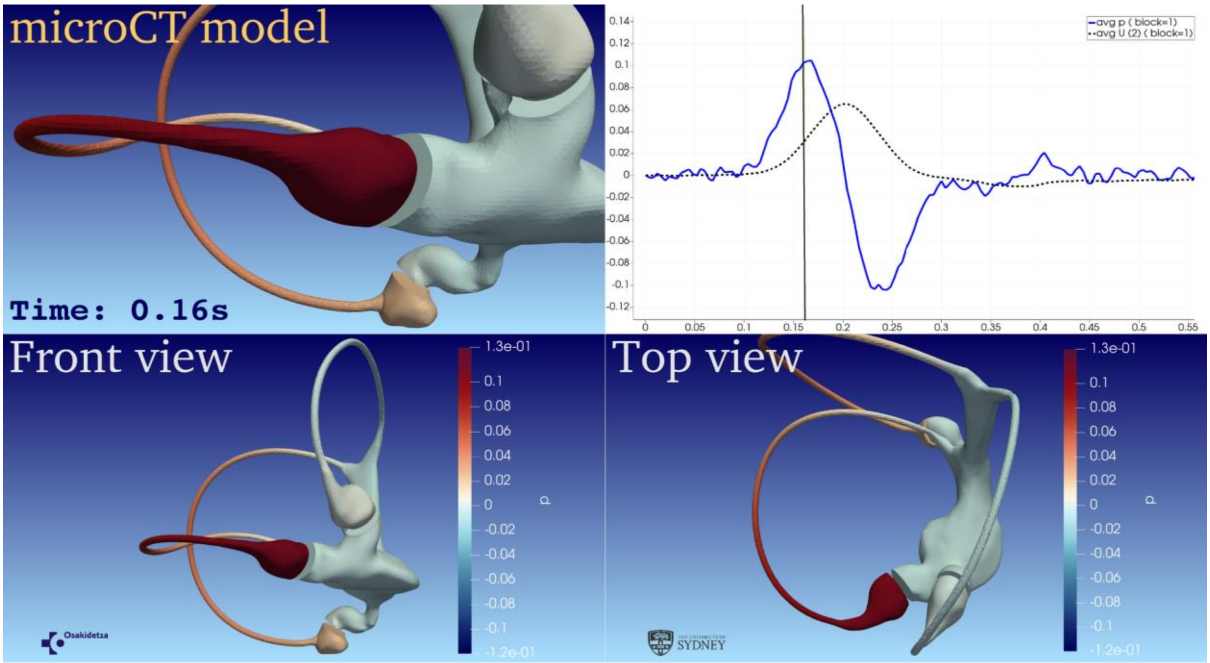

Supplementary Figure 2. To demonstrate that during a horizontal head impulse there is a sizeable stimulus to the posterior semicircular canal, based on microCT evidence of the planes of the human semicircular canals. The plot is a representation of velocity and pressure during the time of a horizontal head impulse to show the stimulus generated in the posterior canal by that horizontal head impulse. Avrg P : is the mean pressure measured at the ampullar wall (corresponding to the cupula) of the horizontal semicircular canal. Avrg U: is the mean magnitude of the 3D vectorial velocity of endolymph, (effectively an endolymphatic “copy” of the head impulse velocity). For this impulse peak head velocity was 249.47 deg/s.

Red shows positive pressure values and blue shows negative pressure values. Note that the excitatory endolymph flow in the horizontal canal is accompanied by an inhibitory endolymph flow in the ipsilateral posterior canal (and thus an excitatory flow in the contralateral posterior canal). Simulation from[44].
